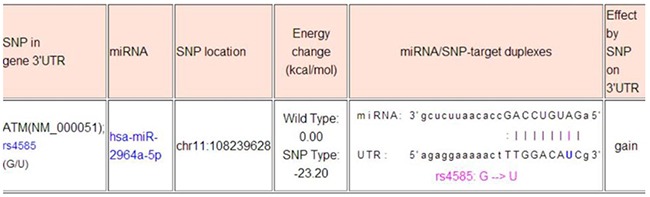# MiR-2964a-5p binding site SNP regulates *ATM* expression contributing to age-related cataract risk

**DOI:** 10.18632/oncotarget.17600

**Published:** 2017-05-03

**Authors:** Han Rong, Shanshan Gu, Guowei Zhang, Lihua Kang, Mei Yang, Junfang Zhang, Xinyue Shen, Huaijin Guan

**Affiliations:** ^1^ Eye Institute, Affiliated Hospital of Nantong University, Nantong, Jiangsu, China; ^2^ Eye Department, The Affiliated Huai’an Hospital of Xuzhou Medical University and The Second People’s Hospital of Huai’an, Huai’an, Jiangsu, China

**Keywords:** microRNA, DNA damage, single-nucleotide polymorphisms (SNPs), ataxia telangiectasia mutated (*ATM*) gene, age-related cataract

## Abstract

This study was to explore the involvement of DNA repair genes in the pathogenesis of age-related cataract (ARC). We genotyped nine single nucleotide polymorphisms (SNPs) of genes responsible to DNA double strand breaks (DSBs) in 804 ARC cases and 804 controls in a cohort of eye diseases in Chinese population and found that the ataxia telangiectasia mutated (*ATM*) gene-rs4585:G>T was significantly associated with ARC risk. An *in vitro* functional test found that miR-2964a-5p specifically down-regulated luciferase reporter expression and *ATM* expression in the cell lines transfected with rs4585 T allele compared to rs4585 G allele. The molecular assay on human tissue samples discovered that *ATM* expression was down-regulated in majority of ARC tissues and correlated with *ATM* genotypes. In addition, the Comet assay of cellular DNA damage of peripheral lymphocytes indicated that individuals carrying the G allele (GG/GT) of *ATM*-rs4585 had lower DNA breaks compared to individuals with TT genotype. These findings suggested that the SNP rs4585 in *ATM* might affect ARC risk through modulating the regulatory affinity of miR-2964a-5p. The reduced DSBs repair might be involved in ARC pathogenesis.

## INTRODUCTION

Age-related cataract (ARC) is a progressive opacification of the ocular lens which can lead to visual impairment and blindness. Based on the opacity location within the lens, ARC can be classified into the following subtypes: cortical cataract (C), nuclear cataract (N), posterior subcapsular cataract (PSC) and mixed cataract (M)[1]. ARC is a multifactorial disease caused by the interactions between genes and environmental factors. Until now, various risk factors like aging, diabetes, gender, sunlight radiation and UV exposure have been associated with cataract development and progression [2].

Currently, the biological processes of ARC are still not fully understood. It is known that external factors such as ionizing radiation and certain chemotherapeutic drugs can generate oxidative stress and induce damages of DNA within the lens epithelium. Disrupted DNA repair genes, which could be caused by gene variation, have been reported to be strongly associated with ARC [3, 4].

Within the mammalian cells, the most severe recorded form of DNA damage is the double-strand DNA breaks (DSB), which usually triggers an irreversible DNA damage response and consequently cell death [5]. It is known that the mechanism of DSB repair (DSBr) includes two distinct pathways: homologous recombination (HR) and non-homologous end joining [6, 7]. HR is partially promoted by the ataxia telangiectasia (*ATM*) gene. The loss of *ATM* gene may lead to low efficiency of HR-mediated DSBr within the cell [8, 9]. Briefly, immediately after the cells have been exposed to radiation, reactive oxidative stress is produced and causes DNA damage. This results in rapid recruitment of repair signal and proteins, as well as an alteration of chromatin structure [10]. When DNA breakage occurs, *ATM* is recruited to the lesion site, thus promoting DSBr and amplifying other DSB signals [11]. Because of *ATM*’s central role in DSBr [12], it is plausible that disrupted *ATM* function might be associated with the occurrence of ARC. Several studies have suggested that specific single-nucleotide polymorphisms (SNPs) within the genes of DSBr pathway may be associated with ARC risk, which include *PARP-1* [13], *ZNF350* [14], *XRCC 1*[15], *WRN* [16] and *EPHA2* [17].

MicroRNAs (miRNAs) are a class of small non-coding RNAs containing approximately 22 nucleotides, which bind to the 3’-untranslated region (3’-UTR) of multiple target mRNAs and block the target translation or initiate a target degradation [18–20]. SNPs present in miRNA-target sites (miRSNPs), and in the 3’-UTR of genes, represent a specific class of functional polymorphisms and may lead to the dysregulation of post-transcriptional gene expression by disrupting regulatory miRNA binding [21]. It has been documented that SNPs in miRNA-target sites confer risky predisposition to complex human diseases, including hypertension [22], cancer [23], Tourette syndrome [24], asthma [25], and Parkinson disease [26].

Recently we have reported several ARC associated SNPs located in intron and coding regions in DNA repair genes such as *WRN* and *BLM* [3]. In this paper, we described a case-control study that aimed to test the relationship between ARC and miRSNPs in the 3’-UTR sequence of *ATM* gene along with its downstream genes *FANCD2* [27], *BRIP1* [28], *TP53* [29], *EPHA2* [30] and *NEIL2* [31]. Subsequently, *in vitro* and *in vivo* assays were conducted in order to clarify the function of the associated SNP.

## RESULTS

The study population was recruited from the epidemiologic survey that comprised 804 patients with ARC and 804 age-, sex- and ethnically-matched healthy control subjects. The general demographic details of the study participants were summarized in Table 1. No statistically significant difference with regard to age and gender was found between case and control populations (*P* > 0.05). Nine SNPs in 3’-UTR region of 7 genes were selected for genotyping, and their basic characteristics and predicted miRNAs binding sites were listed in Table 2. All of the tested SNPs are in HWE in the control population, except *EPHA2* rs1803527 (*P*<0.05) that was excluded from further analysis. We compared the allele frequency between ARC patients and normal controls and found that only *ATM*-rs4585 was associated with ARC (*P*=0.0022, OR=1.24, 95% CI: 1.06–1.40; Table 3 ), and the significance remained after Bonferroni correction (Pa =0.0198).

**Table 1:** Demographic Information of Study Participants

Variable	Control	ARC	*P*
n	804	804	NA
Fasting blood sugar, mean ± SD	69.66±4.51	70.38±7.72	0.735
Age, y, mean ± SD	70.39±5.42	70.87±7.23	0.132
Female, n (%)	455(56.6)	490(60.9)	0.076
Male, n (%)	349(43.4)	314(39.1)	
C, n (%)	0	284(35.32)	NA
N, n (%)	0	349(43.41)	NA
PSC, n (%)	0	33(4.11)	NA
M, n (%)	0	138(17.16)	NA

Abbreviations: C, cortical; N: nuclear; P, posterior subcapsular cataract; M, mixed type

**Table 2:** The Tested 9 SNPs within The 3’-UTR of the Selected 7 Genes in DSBR

Gene Name	SNPs	Assay ID	Nucleotide change	MAF^*^	miRNA binding
*ATM*	rs4585	C___1039793_20	G>T	0.42	hsa-miR-2964a-5p
*FANCD2*	rs7647987	C_189399221_10	A>G	0.15	hsa-miR-4765
	rs3172417	C__27465020_10	C>T	0.10	hsa-miR-516b-5p
*BRIP1*	rs7213430	C___2547428_10	A>G	0.33	hsa-miR-4645-3p
	rs11079454	C____341518_20	T>A	0.44	hsa-miR-101-5p hsa-miR-559 hsa-miR-548b-5p
*TP53*	rs1042522	C___2403545_10	C>G	0.42	hsa-miR-399a-3p
*EPHA2*	rs1803527	C___1472045_20	A>G	0.11	hsa-miR-337-3p
*NEIL2*	rs1534862	C___2716225_10	C>T	0.24	hsa-miR-4289 hsa-miR-544a
*PARP-1*	rs8679	C___9632806_10	T>C	0.07	hsa-miR-4255

*MAF, minor allele frequency in Chinese population.

**Table 3:** Distribution of Minor Allele of Tested 9 SNPs and Their Association with ARC

Gene	SNPs Minor/Major	Control Minor/Major(%)	All ARC Minor/Major(%)	χ^2^	*P*	OR (95%CI)
*ATM*	rs4585 T/G	658/950 (40.92)	744/864 (46.27)	9.35	**0.0022/** **0.0198(Pa)**	1.24 (1.06-1.40)
*FANCD2*	rs3172417 T/C	199/1409 (12.38)	206/1402 (12.81)	0.14	0.71	1.04 (0.84-1.28)
	rs7647987 G/A	206/1402 (12.81)	192/1416 (11.94)	0.56	0.45	0.92 (0.75-1.14)
*BRIP1*	rs7213430 G/A	419/1189 (26.06)	416/1192 (25.87)	0.01	0.90	0.99 (0.84-1.16)
	rs11079454 A/T	797/811 (49.56)	778/830 (48.38)	0.45	0.50	0.93 (0.83-1.10)
*TP53*	rs1042522 G/C	690/918 (42.91)	684/924 (42.54)	0.05	0.83	0.98 (0.86-1.13)
*EPHA2*	rs1803527 G/A	193/1415 (12)	181/1427 (11.26)	0.44	0.51	0.93 (0.75-1.15)
*NEIL2*	rs1534862 T/C	385/1223 (23.94)	373/1235 (23.2)	0.25	0.62	0.9594 (0.82-1.13)
*PARP-1*	rs8679 C/T	100/1508 (6.22)	82/1526 (5.1)	1.88	0.17	0.81 (0.60-1.09)

All HWE (*P* > 0.05) except *EPHA2*-rs1803527 (*P* < 0.05). Pa, *P* value after Bonferroni correction.

We then analyzed the distribution of allele frequencies after stratifying ARC by the subtypes. Briefly, *ATM*-rs4585 was also found to be associated with the cortical and mixed types of ARC (*P* = 0.02, OR=1.25, Table 5 ; *P*=0.009, OR=1.40, Table 4 ), suggesting a risk role for the minor “T” allele in development of the cortical and mixed types of ARC. In addition, *ATM*-rs4585 was marginally associated with the nuclear cataract (*P*=0.03, OR=1.22; Table 4 ). *FANCD2*-rs7647987 was suggestively associated with the nuclear cataract (*P*=0.046, OR=0.75, 95% CI: 0.56-0.99; Table 5 ). From the genetic model analysis, we found that the association of rs4585 with ARC and various subtypes were largely in the recessive model (Table 4), while the association of rs7647987 with nuclear cataract was in the dominant model (Table 5). However, all the associations in genetic model analysis lost significance after using the Bonferroni correction method. This could be explained by the smaller sample size in counting individuals compared to alleles (*P*>0.05; Tables 4, 5). Furthermore, the subtype analysis showed that other SNPs were not associated with ARC subtypes.

**Table 4:** Association between *ATM*-rs4585 and ARC

Allele	G/T	GG+TG/TT
Control, n (%)	950(59.08)/658(40.92)	664(82.59)/140(17.41)
General ARC, n (%)	864(53.73)/744(46.27)	621(77.24)/183(22.76)
*P*/Pa OR (95%CI)	**0.0022/0.0198** 1.24 (1.06-1.40)	0.07/0.63 1.40 (1.09-1.79)
Cortical cataract, n (%)	304(53.52)/264(46.48)	211(77.01)/63(22.99)
*P*/Pa OR (95%CI)	**0.02**/0.18 1.25 (1.03-1.52)	**0.049**/0.45 1.35 (1.00-1.88)
Nuclear cataract, n (%)	379(54.3)/319(45.7)	269(77.08)/80(22.92)
*P*/Pa OR (95%CI)	**0.03**/0.27 1.22 (1.02-1.45)	**0.03**/0.27 1.41 (1.04-1.92)
Posterior subcapsular cataract, n (%)	41(62.1)/25(37.9)	39(90.70)/4(9.30)
*P*/Pa OR (95%CI)	0.62/5.58 0.88 (0.53-1.46)	0.21/1.89 0.65 (0.23-1.89)
Mixed type of cataract, n (%)	140(50.72)/136(49.28)	102(73.91)/36(26.09)
*P*/Pa OR (95%CI)	**0.009**/0.081 1.40 (1.09-1.81)	**0.02**/0.18 1.67 (1.10-1.55)

**Table 5:** Association between *FANCD2*-rs7647987 and ARC

Allele	G/A	GG /AG+AA
Control, n (%)	1402(87.19)/206(12.81)	610(75.87)/194(24.13)
General ARC, n (%)	1416(88.06)/192(11.94)	628(78.11)/176(21.89)
*P*/Pa OR (95%CI)	0.45/4.05 0.92 (0.75-1.14)	0.29/2.61 0.88 (0.70-1.11)
cortical cataract, n (%)	450(79.23)/118(20.77)	219(77.11)/65(22.89)
*P*/Pa OR (95%CI)	0.12/1.08 0.83 (0.66-1.05)	0.67/6.03 0.93 (0.68-1.29)
Nuclear cataract, n (%)	629(90.11)/69(9.89)	284(81.38)/65(18.62)
*P*/Pa OR (95%CI)	**0.046**/0.45 0.75(0.56-0.99)	**0.04**/0.36 0.72 (0.53-0.99)
Posterior subcapsular cataract, n (%)	53(80.30)/13(19.70)	23(69.7)/10(30.3)
*P*/Pa OR (95%CI)	0.10/0.90 1.67 (0.89-3.12)	0.42/3.78 1.37 (0.64-2.92)
Mixed type of cataract, n (%)	237(85.87)/39(14.13)	102(73.91)/36(26.09)
*P*/Pa OR (95%CI)	0.55/4.95 1.12 (0.77-1.62)	0.62/5.58 1.11 (0.73-1.68)

**Figure 1:**
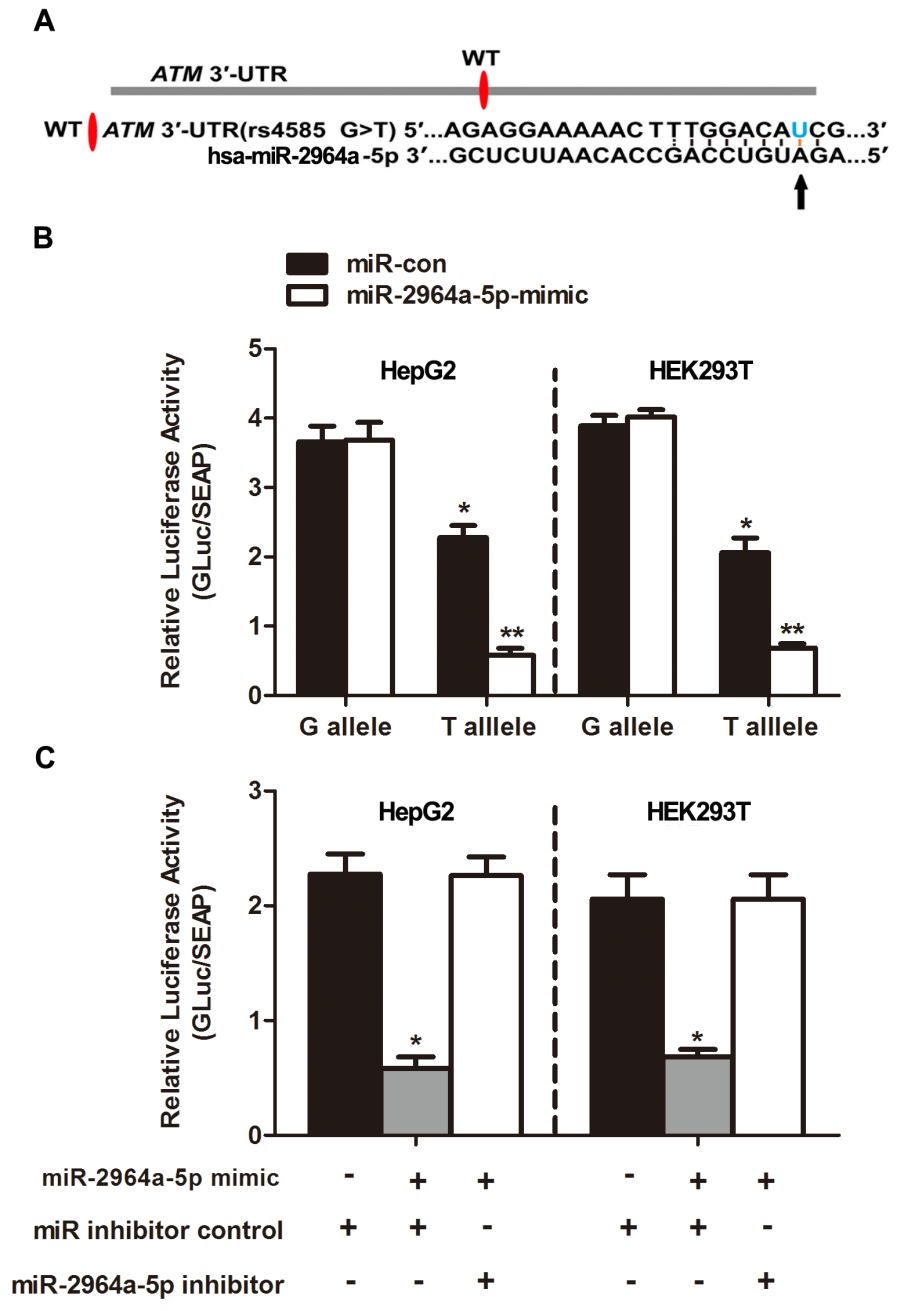
The effect of miR-2964a-5p on miRSNP rs4585 **(A)** The schema showed predicted binding site of miR-2964a-5p in the 3’-UTR of the *ATM*. **(B)** MiR-2964a-5p mimics or miR-con was co-transfected with the reporter constructs containing G allele or T allele into HEK293T or HepG2 cells. *: *P*<0. 05, compared with G allele group; **: *P*<0. 01, compared with miR-con group. **(C)** MiR-2964a-5p mimics, inhibitors or miR inhibitor control was co-transfected with the reporter gene containing T allele into HepG2 or HEK293T cells. *: *P*<0. 01, compared with miR inhibitor control group.

Using the miRNA-target prediction tools, we determined that the *ATM* 3’-UTR harbored a putative miR-2964a-5p miRNA binding site (Figure 1A). In accordance with the previous predictions, the relative luciferase activity was lower in the presence of the “T” than of the “G” allele in both HepG2 (*P*<0.01) and HEK293T (*P*<0.01) cells, and without an addition of external miRNA (Figure 1B). Moreover, synthesized miR-2964a-5p mimics significantly down-regulated luciferase reporter expression in both cell lines transfected with rs4585 “T” allelic reporter constructs compared to miRNA-control (miR-con), whereas there was no such suppression of luciferase activities in both cell lines transfected with the “G” allelic reporter constructs by the miRNA mimics (Figure 1B). Notably, the reporter expression was lower in the presence of the “T” than of the “G” allele, which might be explained by the existence of endogenously miRNAs. In addition, co-transfection with miR-2964a-5p inhibitors, chemically synthesized oligonucleotides that neutralize endogenous miRNA, significantly recovered the activities of reporter gene with the rs4585 “T” allele (Figure 1C), while no change was observed for reporter gene expression with the “G” allele treated with the inhibitors (data not shown).

**Figure 2:**
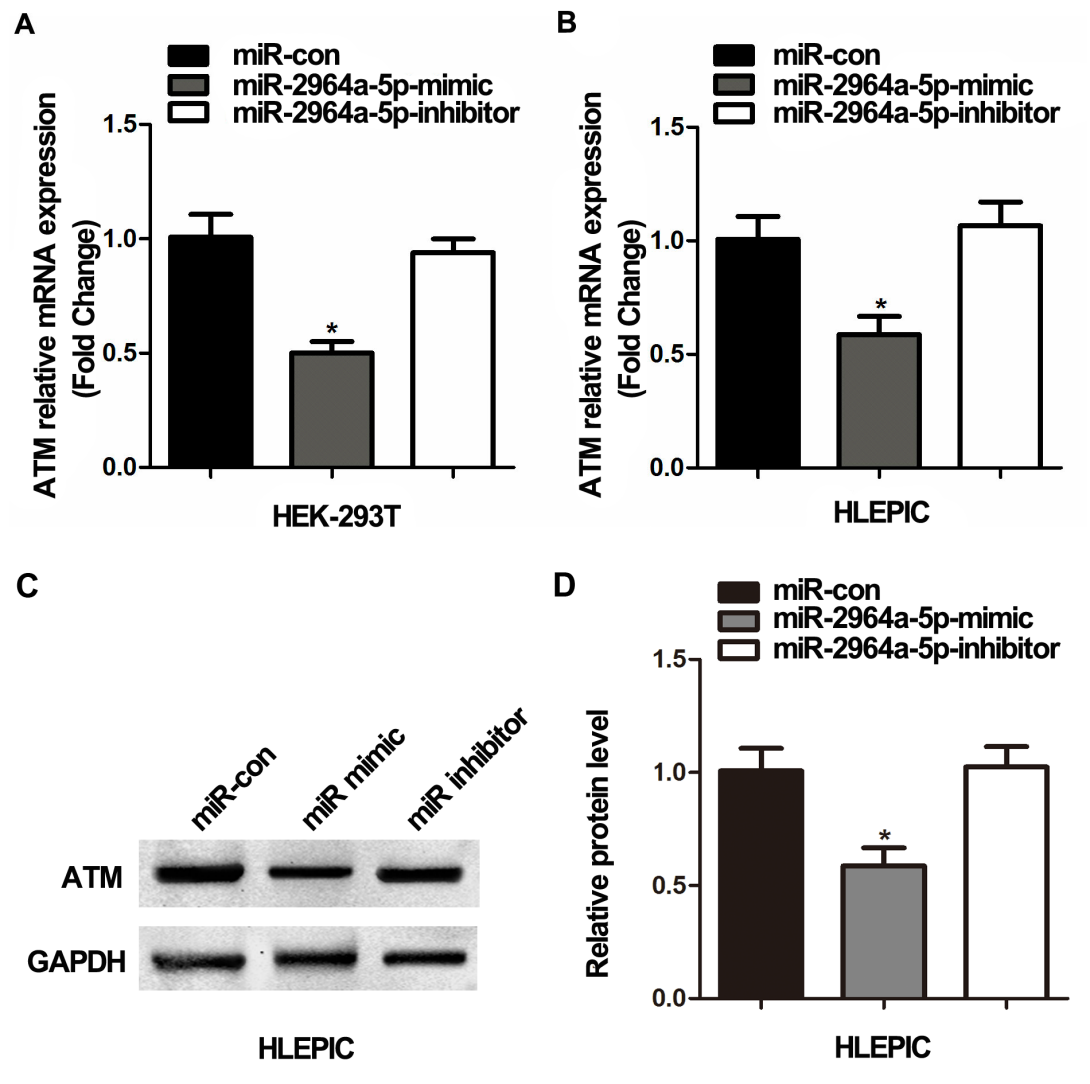
The correlation of SNP rs4585 with *ATM* expression *in vitro* **(A)** Analysis of *ATM* mRNA expression in HEK293T cells (TT) transfected with miR-2964a-5p mimics and inhibitors. **(B)** Analysis of *ATM* mRNA levels in HLEPIC-LECs (TT) transfected with the miR-2964a-5p mimics and inhibitors. **(C)** Western blot analysis and quantification (D) of *ATM* expression in HLEPIC-LECs (TT) transfected with the miR-2964a-5p mimics and inhibitors. *: *P*<0.01, compared with control group.

Considering the direct association found between miR-2964a-5p and *ATM* 3’-UTR (results found by using luciferase assay), we further investigated whether miR-2964a-5p alone could inhibit *ATM* expression in cell lines carrying the TT genotype. We measured *ATM* expression directly, after transfecting HEK293T cells (TT genotype) with miR-2964a-5p mimics and miR-2964a-5p inhibitors. As shown in Figure 2A, the *ATM* expression decreased when mimics were added, while the suppression of *ATM* expression was abolished as the inhibitors were co-transfected. Furthermore, we validated if miR-2964a-5p can down-regulate the expression of *ATM* in LEC lines, miR-2964a-5p mimics or inhibitors were transfected into HLEPIC-LECs (TT genotype), similar results were observed change (Figure 2B, 2C, 2D).

**Figure 3:**
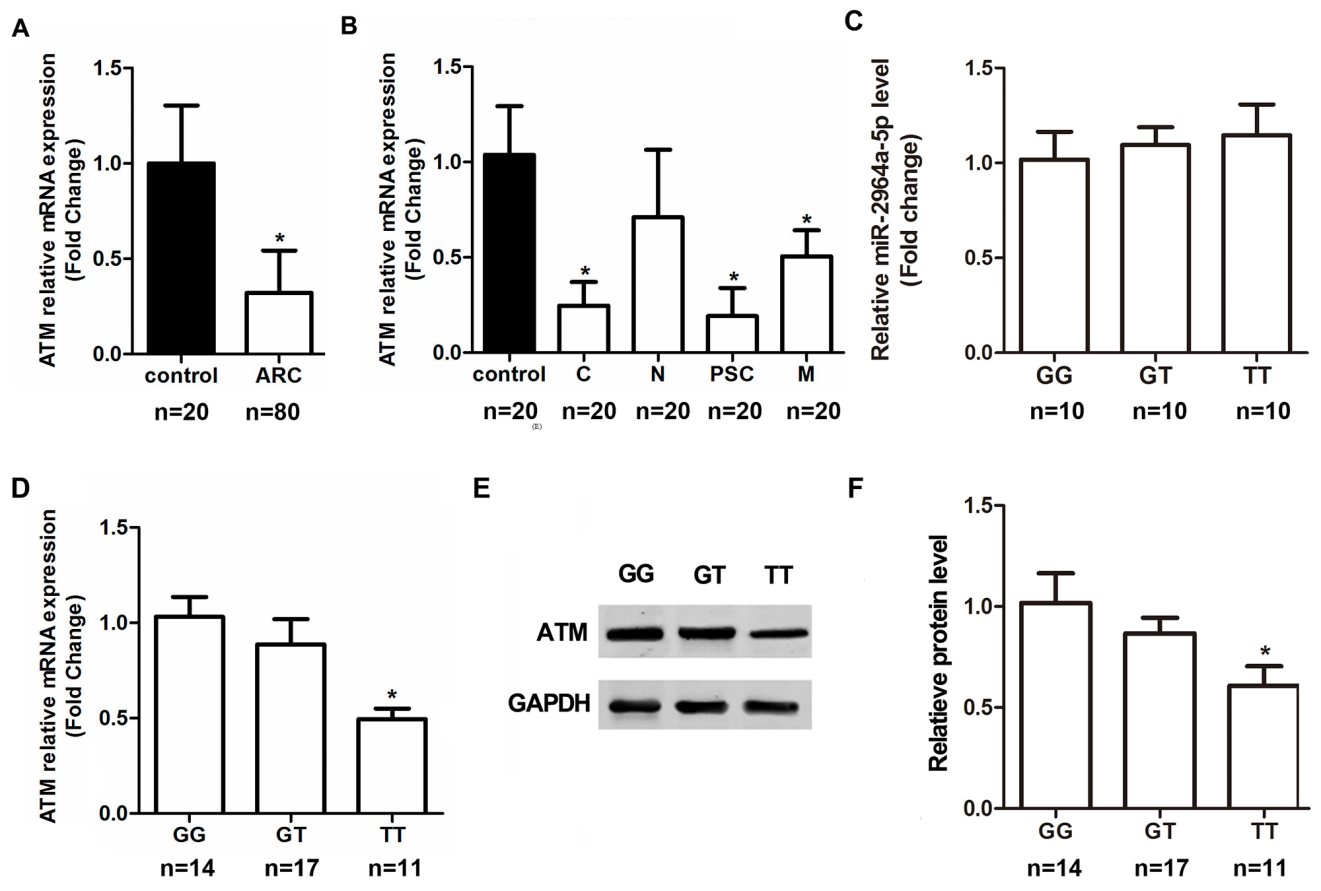
The correlation of SNP rs4585 with *ATM* expression in anterior capsules **(A)** *ATM* mRNA expression was lower in ARC group than the non-ARC group regardless of genotypes. **(B)** After stratifying ARC by the subtypes, *ATM* mRNA levels were markedly decreased in the cortical, posterior subcapsular and mixed types of ARC regardless of genotypes. **(C)** Levels of miR-2964a-5p in anterior capsules of three genotypes were similar in ARC, *P*=0.2190. **(D)** *ATM* mRNA levels in ARC were lower in TT group than the GG or GT group. **(E)** Western blot analysis and quantification **(F)** of *ATM* expression in samples from GG, GT or TT group of ARC. *: *P*<0.01.

In addition, we measured the *ATM* expression levels within LECs. The *ATM* mRNA expression was lower in the ARC group compared to the Control group regardless of genotypes (Figure 3A), significant mRNA expression was observed in the cortical, posterior subcapsular and mixed types of ARC (Figure 3B). Moreover, a number of anterior capsule samples, with different genotypes of rs4585, proved that real biological effects resulted from the allele difference. The results from ARC individuals showed that “TT” genotype of rs4585 (n = 11) has lower level of *ATM* mRNA (Figure 3D) and protein expression (Figure 3E, F) compared to other two genotypes ( “TT” versus “GG”, *P*< 0.01; “TT” versus “GT”, *P*< 0.01; Figure 3D), although no significant differences were found between “GG” (n = 14) and “GT” (n = 17) genotypes ( “GG” versus “GT”, *P*> 0.05). In order to further investigate the possible regulation of *ATM* mRNA by miR-2964a-5p, and the expression in a genotype-dependent manner, 30 tissue samples were used to detect the expression level of miR-2964a-5p. However, miR-2964a-5p expression was not influenced by *ATM* genotype (Figure 3C). Thus, by conclusion, our results surrested that variant alleles in the *ATM* 3’-UTR affect the binding ability of miR-2964a-5p to *ATM*, but not the abundance of miR-2964a-5p itself.

**Figure 4:**
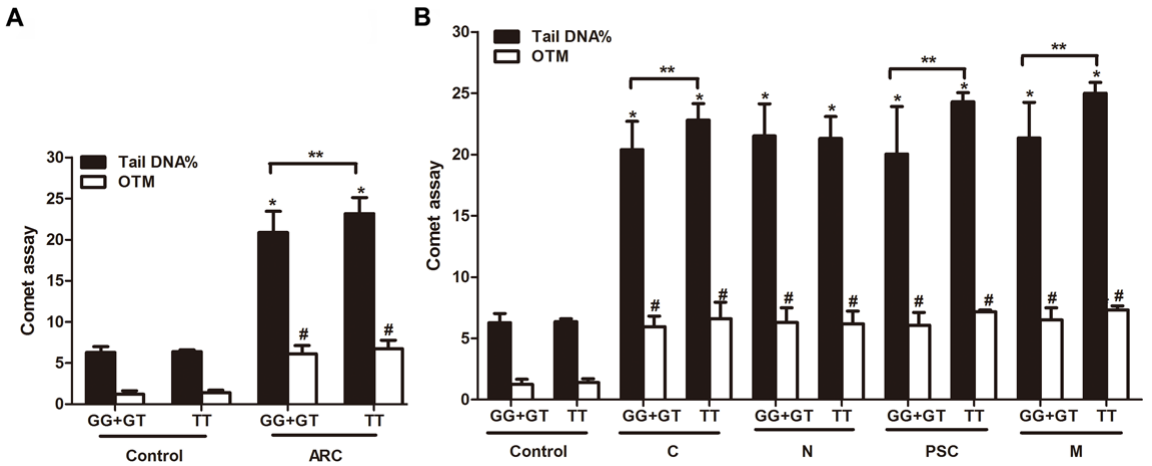
The correlation of rs4585-T with DNA breaks and ARC risk (**A**) ARC had more DNA breaks than the control and TT carriers had more DNA damage than GG+GT carriers in ARC. (**B**) TT carriers had more DNA damage than GG+GT carriers in cortical, posterior subcapsular and mixed cataract patients, in addition to nuclear cataracts. *: *P*< 0.01, compared with control group of GG+GT genotype (n= 27), ^#^: *P*< 0.01, compared with control group of TT genotype (n=5), **: *P*< 0.05, compared with corresponding ARC patients with GG+GT carriers.

The peripheral lymphocytes DNA breaks analysis showed that the percentage of DNA in the tail of Comets (the tail DNA%) and the value of the Olive Tail Moment (OTM) were significantly different in ARC group (Figure 4A) or all ARC subtypes group (Figure 4B) compared to the non-ARC controls (*P*< 0.001). We also found that there was a good correlation of DNA breaks with the rs4585 genotypes. Homozygous “T” allele carriers of *ATM*-rs4585 had more DNA breaks compared to “G” allele carriers in ARC, cortical, posterior subcapsular and mixed cataract patients (*P*< 0.05; Figure 4A and B). We also stratified the results by males and females and did not find the difference in the DNA breaks between the genders (*P*> 0.05, data not shown).

## DISCUSSION

DNA damage is considered to be a crucial contributor to the formation of ARC, and its timely repair is essential for maintaining the lens’ transparency [32]. *ATM* gene has been shown to play an important role in DNA repair. Previous studies have reported correlations between DNA repair capacity and the regulation of *ATM* expression [33, 34]. It has been demonstrated that the aberrant overexpression of miR-421 may down-regulated *ATM*, therefore, can lead to SKX squamous cell carcinoma [35].In addition, it has been shown that miR-203 may be responsible for *ATM* down-regulation in breast cancers [12].

In this case-control study, we found that only rs4585 in *ATM* was solidly associated with an increased risk of ARC. Furthermore, we have characterized rs4585 as a unique SNP that conferred a genetic effect on gene regulation by miRNA binding. MiRNAs are short genetic sequences with the ability to regulate gene expression by binding to the 3’-UTR of the target gene [36]. Different expression profiles of miRNAs were identified in the central epithelium of transparent and cataractous human lenses [37]. Our results have suggested that miR-2964a-5p binds more tightly to “T” allele of rs4585 and represses *ATM* expression more strongly compared to “G” allele. This data was validated by using a luciferase reporter gene method, as well as *in vitro* assays. Most importantly, our data have suggested that patients carrying the rs4585 “T” allele in the 3’-UTR of *ATM*, show a higher risk for ARC development, and exhibit lower *ATM* expression within the lens tissues of most of ARC subtypes, with an exception of the nuclear cataract subtype.

In the eye lens, oxidative stress may induce various types of DNA damage, which consequently, may cause cataract [38–40]. In our previous study [41], we found that DNA damage in LECs was positively related to that in peripheral lymphocytes. In addition, different DNA damage of peripheral lymphocytes was detected in individuals with different genotypes [42]. This data support a notion that systematic oxidative stress might attribute to the local lesion of the lens. In this study, we found that the extent of DNA breaks in peripheral lymphocytes, analyzed using the Comet assay, was significantly higher in all ARC subtypes, regardless of the genotype. These results were in line with the results obtained by Ates et al. which found an elevated levels of 8-OH-Gua, a marker of oxidative DNA damage, in the leukocytes of patients with cataract [43]. However, the control lymphocytes, which presumably represent a mixture of genotypes, had dramatically lower breakage than the cataract patients in our study. This data has suggested that there is another factor involved in this mechanism, and that presumably has a larger effect on DNA breakage compared to rs4585 genotype. However, the measurement of more subtle oxidative lesion in lens tissues and the characterization of its correlation with the SNP genotypes are still warranted in our future studies.

Recently, a subset of the long non-coding RNAs (lncRNAs) has been reported to interact with miRNAs by complementary sequence, and to act as a miRNA decoy or sponge, which then indirectly influences the miRNA regulation of other protein-coding genes. It has been also demonstrated that, the presence of SNPs in miRNA target sites of lncRNAs, may impact the expression of lncRNA by influencing the miRNA-lncRNA interactions, therefore promoting or inhibiting the occurrence and development of diseases. For example, Jing et al. have found a functional polymorphism in lnc-LAMC2-1:1which conferred risk of colorectal cancer by affecting miRNA binding [44]. Tao et al. have demonstrated that the function of the SNP rs145204276 might be mediated by affecting methylation status of the GAS5 promoter and subsequently its transcriptional activity thus serving as a potential therapy target for hepatocellular carcinoma [45]. Moreover, Ling et al. have identified a colon cancer susceptibility SNP rs6983267 which may exert its function by regulating the expression of lncRNA CCAT2 [46]. Therefore, we hypothesized that there may exist functional SNPs within lncRNAs, especially in miRNA:lncRNA binding sites, which are associated with the occurrence of ARC. Further studies are necessary to investigate these loci.

To conclude, our results have suggested that miRSNP rs4585 “T” allele of *ATM* gene may be associated with increased risk for ARC in Han Chinese population. The mechanism underlying may be that the “T” variant of rs4585 enhanced miR-2964a-5p regulatory affinity to the 3’-UTR of the *ATM* gene, which in turn may disrupt the post-transcriptional regulation of the *ATM* gene, thus contributing to ARC.

## METHODS

The research acted in adherence to the tenets of the Declaration of Helsinki. All participants signed the respective informed consent forms. The study was approved by the Ethics Committee of Affiliated Hospital of Nantong University.

This study was a part of population-based epidemio-logic survey of the Jiangsu Eye Study [3, 16, 42, 47]. The sampling method and inclusion/exclusion criteria for this on-going cohort were in consistent with our previous study [3]. Briefly, the cataract was defined as opacification of ocular lens and best-corrected visual acuity (BCVA) >20/40. Lens opacities were graded according to the Lens Opacities Classification System III (LOCSIII) [48] in 0.1 unit steps for each opacity up to a maximum of 6.9 for nuclear opacities, and 5.9 for cortical and posterior subcapsular. The presence of more than one cataract type in at least one eye, or different pure types in both eyes were classified into the mixed type [49]. Patients with cataracts due to trauma, diabetes mellitus, uveitis, glaucoma and other causes were excluded from the study. As a result, there were 834 ARC patients included in this study. There were 30 cases failed in DNA extraction and genotyping. We finally examined 804 ARC patients. The non-ARC controls were also selected from the epidemiologic study population without cataracts. After matching for age and sex, 804 individuals were included as normal controls.

In order to measure mRNA, protein and miRNA levels in lens tissues of ARC patients and normal controls, additional 80 ARC patients (cortical=20, nuclear=20, posterior subcapsular=20 and mixed=20) from our clinic (Affiliated Hospital of Nantong University) were included for lens epithelial cells (LECs) collection during the surgery. The control transparent lens was obtained from 20 age-matched subjects who had lens extraction during epiretinal membrane removal. All those clinical cases received phacoemulsification and intraocular lens implantation. There were no statistically significant differences between the two groups regarding the age (*P*>0.05).

### Selection of miRSNPs and SNP genotyping

We used database and literature search to screen DSBR genes and focused on *ATM* pathway for this project. A 3’-UTR dataset and a miRNA target dataset of human genes were obtained from the databases of NCBI dbSNP BUILED129 (http://www.ncbi.nlm.nih.gov/SNP) and the International HapMap Project (http://hapmap.ncbi.nlm.nih.gov/). The online softwares miRNASNPV2.0 (http://www.bioguo.org/miRNASNP2/geneTargets.php) and TargetScan V5.1 (http://targetscan.org/) were used to predict the possible miRNA binding-sites in the 3’-UTR. We excluded SNPs with minor allele frequencies (MAF) less than 10% in the HapMap CHB population. A further selection criterion was a SNP with r^2^ value≤0.8 in comparing with its neighboring loci to exclude strong linkage disequilibrium between adjacent variants. The selected SNPs in 7 DSBR genes are listed in Table 2.

Peripheral venous blood was collected in an EDTA anticoagulation tube. Genomic DNA was isolated from leukocytes by the phenol-chloroform method. Genotyping of all SNPs were conducted with a commercial gene expression assay (TaqMan Assay; Applied Biosystems, Foster City, CA, USA), as described in our previous publications [50, 51].

### LECs collection and DNA/RNA/protein isolation

The LECs were collected by anterior continuous curvilinear capsulorhexis during cataract surgery. The LECs were collected from transparent anterior capsulorhexis of controls and opaque anterior capsulorhexis of ARC patients. The sample was rapidly frozen in liquid nitrogen, and then stored at −80°C for later extraction of genomic DNA, RNA and protein.

### Cell culture and co-transfection

Human lens epithelial cell line (HLEPIC) and human embryonic kidney cell line (HEK-293T) and human hepatoblastoma cell line (HepG2) were purchased from American Type Culture Collection (ATCC; Rockville, MD, USA). Cells were cultured in Dulbecco’s modified Eagle’s medium (DMEM; Invitrogen, CA, USA) supplemented with 10% fetal bovine serum (FBS; Invitrogen, CA, USA) at 37 °C in a humidified incubator with 5% CO_2_. Low passage cells were used in experiments 24 hours before transfection, cells were plated onto a 24-well plate at a density of 2×10^5^ cells/well. The transfection was conducted when cells reached 80% confluence.

HEK-293T and HepG2 cells were transiently co-transfected with reporter plasmids and miRNA mimics or inhibitors (Ribobio, Guangzhou, China). HEK-293T and HLEPIC cells were only transfected with miRNA mimics or inhibitors for qRT-PCR or Western blot assays. The miRNA inhibitors were chemically-modified and optimized complementary single stranded nucleic acids designed to specifically target the miRNA and knockdown individual miRNA molecules.

### Plasmids construct

The luciferase assay was used as the reporter system to assess functional consequences for the significant 3’-UTR SNP rs4585. We first synthesized oligonucleotides that contain 201 bp surrounding SNP rs4585 in three tandem copies and used the restriction enzymes EcoRI and XhoI for cloning sites. The cloning sites exploited for generation of reporter construct (rs4585) were as follows: tggtcattatagtatatgcctaaaatgtatgcacttaggaatgctaaaaatttaaatatggtctaaagcaaataaaagcaaagaggaaaaactttggacaG/Tcgtaaagactagaatagtcttttaaaaagaaagccagtatattggtttgaaatatagagatgtgtcccaatttcaagtattttaattgcaccttaatgaa. The oligonucleotides were cloned into the secreted Gaussia luciferase (GLuc) reporter gene system, driven by SV40 promoter for expression in mammalian cells. A secreted Alkaline Phosphatase (SEAP) reporter, driven by a CMV promoter, was also cloned into the same vector (pEZX-MT05; GeneCopoeia, Rockville, USA) and served as the internal control. The minor T allele of rs4585 was similarly constructed. All constructs were confirmed by DNA sequencing.

### Luciferase reporter assay

For luciferase reporter assays, HEK-293T cells and HepG2 cells were co-transfected with 600 ng of *ATM* 3’-UTR luciferase plasmid (pEZX-MT05) and miRNA mimics/inhibitors or control miRNA mimic, using Lipofectamine 2000 (Invitrogen, CA, USA). Gaussian luciferase and alkaline phosphatase activities were measured by luminescence in conditioned medium 48 hours after transfection using these secreted-pair dual luminescence kit (GeneCopoeia, Rockville, USA). Gaussian luciferase activity was normalized to alkaline phosphatase activity. Each sample was measured in triplicate using the Glomax Luminometer (BioTek, Beaumont, Texas, USA).

### RNA isolation and cDNA preparation

Total RNA was isolated from the frozen LEC tissues or cell culture using TRIzol reagent (Invitrogen, CA, USA) and cDNAs were synthesized using Prime Script® RT reagent Kit (Takara, Dalian, China).

### Quantification of mRNA and miRNA expression

TaqMan gene expression assay probes (Applied Biosystems, USA) were used for *ATM* mRNA quantification (assay ID: Hs01112355_g1). Human beta-actin (assay ID: Hs01060665_m1) was chosen as housekeeping gene for normalization. Measurement of miRNA levels in lens anterior capsules samples were determined by qRT-PCR with primers (GeneCopoiea, Rockville, USA) specific for candidate miRNA. qRT-PCR was carried out using the All-in-OneTM miRNA qRT-PCR Detection Kit (GeneCopoiea, USA). U6 small nuclear RNA (snRNA) was selected as the endogenous control. The catalog numbers of All-in-One miRNA qPCR Primers were as follows: hsa-miR-2964a-5p∼HmiRQP1876; snRNA U6∼HmiRQP9001.qRT-PCR was performed using ABI 7500 real time PCR system (Applied Biosystems, USA). The fold change of gene or miRNA expression was determined using the comparative CT (2^-ΔΔCT^) method.

### Western blot assay

The protein of LECs and HLEPIC were extracted separately in lysis buffer (1 M Tris-HCl at pH 7.5, 1% Triton X-100,1% Nonidet p-40, 10% SDS, 0.5% sodium deoxycholate, 0.5 M EDTA, 10 μg/ml leupeptin, 10 μg/ml aprotinin, and 1 mM phenylmethylsulfonyl fluoride [PMSF]). Equal amounts of proteins were size fractionated by sodium dodecyl sulfate-polyacrylamide gel electrophoresis on 15% polyacrylamide gels. Proteins were then transferred onto polyvinylidene difluoride filter membranes (Millipore, Bedford, MA, USA). The blocked membrane was then incubated with mouse anti-human-ATM (Sigma) and mouse antihuman-GAPDH (1:1000; Abcam, Cambridge, UK) at 4 °C for 12 hours. After washing, the membrane was incubated with an alkaline phosphatase-conjugated goat anti-mouse IgG antibody (1:2000; Santa Cruz) for 2 hours. An enhanced chemiluminescence detection system was used to read the Western signals (Pierce Company, USA).

### Comet assay

The Comet assay (also known as the Single Cell Gel Electrophoresis assay) is a sensitive technique for the detection of DNA damage at the level of an individual cell. We collected fresh blood from 106 ARC patients (cortical=44, nuclear=30, posterior subcapsular=10 and mixed=22) and 32 age and sex matched normal controls in the Jiangsu Eye Study for Comet assay that measures DNA breaks of lymphocytes and the genotyping. The modified Comet assay for lymphocytes and data analysis was carried out according to the method described by Zhang et al [41]. OTM, defined as the product of the distance between the barycenters of the head and tail by the percentage of DNA in the tail of the Comet-like images, was used to evaluate the extent of DNA damage (DNA breaks) in individual cells.

### Statistical analysis

Statistical analyses were performed with a commercial statistical software program (Stata 8.0; Stata Corp, College Station, TX). The X^2^ test was used to test the association between the alleles frequencies of all ARC patients and healthy controls and various ARC subtypes. In addition, X^2^ test was used to analyze\estimate odds ratios (OR) and 95% confidence interval (CI), as well as Hardy-Weinberg Equilibrium (HWE) of genotype distributions. If positive association was found in the initial allele analysis, Bonferroni correction was performed. Various genetic model analyses were performed to characterize the association as dominant (heterozygote and homozygote versus wild type), recessive model (homozygote versus wild type and heterozygote), additive model (homozygote versus heterozygote versus wild type), and heterozygote advantage model (heterozygote versus homozygote and wild type). We only present the most significant model in the results. In addition, all *in vitro* experiments were done independently and in triplicated. Statistical comparisons of the average values of 2 groups were performed using the Student t test, or assessed by one-way analysis of variance (ANOVA) when more than two groups were analyzed, with a *P* value of >0.05 considered significant.

### Note

The web-based software “miRNA SNP” (http://www.bioguo.org/miRNASNP2/geneTargets.php) that was used in the study design has been removed by the owner and is no longer available. The minimal free energy (MFE) analysis by paring the sequences of hsa-miR-2964a-5p (hsa-miR-219b-5p, identical miRNA with different annotation) and rs4585, the major discovery of this work, indicates -18.9 kcal/mol in MFE (https://bibiserv.cebitec.uni-bielefeld.de/sessionTimeout.jsf). The common agreement in the field is that -20 kcal/mol in MFE is the cut-off for a suitable binding potential, therefore -18.9 kcal/mol in our case indicates a suitable binding potential between the hsa-miR-2964a-5p and DNA sequence around rs4585. (see below).

**Table:**